# Lessons Learnt from the Belimumab Trials in Systemic Lupus Erythematosus

**DOI:** 10.3390/ijms27010037

**Published:** 2025-12-19

**Authors:** Leonardo Palazzo, Alexander Tsoi, Dionysis Nikolopoulos, Ioannis Parodis

**Affiliations:** 1 Division of Rheumatology, Department of Medicine Solna, Karolinska Institutet, Karolinska University Hospital, Center for Molecular Medicine (CMM), SE-171 76 Stockholm, Sweden; 2 Department of Rheumatology, Faculty of Medicine and Health, Örebro University, SE-701 82 Örebro, Sweden

**Keywords:** systemic lupus erythematosus, belimumab, BLISS, post hoc analysis, outcome measures, belimumab, B cell therapies

## Abstract

Belimumab, a human monoclonal antibody that works against B-cell activating factor (BAFF), has significantly advanced the management of systemic lupus erythematosus (SLE). Beyond the initial Phase III randomised controlled trials (RCTs) that demonstrated efficacy for belimumab as an add-on to non-biological standard therapy (ST) along with a favourable safety profile, more than 50 post hoc analyses of RCT data have provided additional insights into its clinical utility. These analyses have shown uniformly that belimumab increases the likelihood of achieving meaningful reductions in disease activity, sustained low disease activity, and improved health-related quality of life (HRQoL) outcomes, with more pronounced benefits in serologically active SLE. Studies focusing on organ-specific manifestations revealed that belimumab confers benefits across multiple SLE facets, with prominent effects on musculoskeletal and mucocutaneous symptoms. Along the same lines, post hoc analyses of the BLISS-LN trial demonstrated benefit from belimumab regarding multiple renal outcomes, including reduced renal flare rates, improved glomerular filtration rate, and improved histological findings in repeat kidney biopsies. Long-term extension studies and real-world evidence confirm its durable efficacy and safety, with continued reductions in overall disease activity, glucocorticoid use, and healthcare resource utilisation over several years. By exploring different efficacy endpoints, person-centred outcomes, disease trajectories, and characteristics across organ manifestations, this body of post-marketing evidence has not only enhanced our understanding of belimumab use in SLE but also constitutes a comprehensive framework for future clinical trial design and development of novel therapeutic strategies. The present review summarises key findings of post hoc analyses of RCTs and observational studies of belimumab.

## 1. Introduction

Belimumab is a monoclonal antibody that targets the B-lymphocyte stimulator (BLyS), a key factor in B cell survival and a contributor to the pathogenesis of systemic lupus erythematosus (SLE) [1]. It was first approved in 2011 for the treatment of moderately to highly active SLE based on the positive outcomes of two pivotal Phase III clinical trials: BLISS-52 [2] and BLISS-76 [3]. These studies demonstrated the efficacy and favourable safety of intravenous (i.v.) belimumab as an add-on therapy to non-biological standard therapy (ST) in patients with active, autoantibody-positive SLE. Following these initial trials, three additional studies (BLISS-NEA [4], EMBRACE [5], and BLISS-SC [6]) were conducted to further evaluate its effectiveness across more diverse patient populations and to assess the efficacy and convenience of the subcutaneous (s.c.) administration. Collectively, these trials enrolled over 3500 patients, making this clinical programme one of the largest and most comprehensive datasets in SLE research. This extensive dataset has not only facilitated post hoc studies to substantiate the clinical efficacy of belimumab but has also provided a valuable resource for broader research efforts in the field beyond belimumab. In 2020, the BLISS-LN study led to an expanded indication for belimumab, with approval of its use as an add-on therapy for active lupus nephritis, signifying a substantial advancement in the management of this severe manifestation of SLE [7].

Beyond the primary efficacy and safety outcomes, over 50 post hoc analyses have been conducted using data from the clinical trials of belimumab. These studies have enhanced our understanding of clinical outcomes in SLE as well as the role of belimumab in the management of the disease, e.g., by exploring ways to optimise the use of outcome measures used in clinical trials [8,9,10,11,12,13,14,15,16,17], the importance of patient-reported health-related quality of life (HRQoL) as a part of the disease management [18,19,20,21,22,23,24,25,26,27,28,29,30,31,32,33,34], the trajectory and characteristics of specific SLE manifestations [35,36,37,38,39,40,41], indicators of disease flares [42,43,44,45,46,47], and predictors of long-term outcomes [48,49,50]. Herein, we discuss and summarise key findings from post hoc analyses of the main clinical trials of belimumab that have been published to date aiming to offer a clinically oriented synthesis that extends the information available from previous reviews and meta-analyses [51,52,53,54] to support decision-making in routine SLE care.

## 2. The Belimumab Trials

A summary of the main clinical trials conducted to assess safety and efficacy of belimumab is provided in Table 1.

### 2.1. First Phase II Trial

The first Phase II randomised, double-blind, placebo-controlled trial evaluated belimumab (1, 4, or 10 mg/kg every 4th week) versus placebo in 449 SLE patients over 52 weeks [55]. While the trial did not meet its two co-primary efficacy endpoints (Reduction of the Safety of Estrogens in Lupus Erythematosus: National Assessment version of the Systemic Lupus Erythematosus Disease Activity Index (SELENA-SLEDAI) [58] at week 24, and time to first flare), it provided insights into the biological activity of belimumab and its therapeutic potential. The drug induced greater reductions in CD20+ B cells and anti-double stranded DNA antibody (anti-dsDNA) levels compared to placebo. Post hoc analyses revealed that serologically active patients (defined as anti-nuclear antibody titre > 1:80 and/or anti-dsDNA level > 30 IU/mL; 71.5% of the study population) showed significantly better responses across multiple measures, including SELENA-SLEDAI, physician’s global assessment (PGA), and 36-item Short Form Health Survey (SF-36) physical component summary (PCS) scores [59]. The safety profile of belimumab was comparable to that of the placebo. This study informed the design of subsequent Phase III trials by identifying serologically active patients as the optimal target population [2,3,4,5,6]. Moreover, it informed the generation of the Systemic Lupus Erythematosus Responder Index (SRI), which is currently one of the most commonly used composite endpoints in Phase III clinical trials [60].

### 2.2. BLISS-52 and BLISS-76

Following the Phase II trial, two pivotal Phase III trials were conducted. BLISS-52, conducted primarily in Asia, South America, and Eastern Europe, enrolled 867 patients with SLE, and was the first to demonstrate significant efficacy of belimumab in reducing disease activity when added to ST [2]. Participants received belimumab 1 mg/kg (n = 289), 10 mg/kg (n = 290) or placebo (n = 288) plus ST for 52 weeks. The primary endpoint was the SRI-4 [54] at week 52, and a dose-dependent response was noted with 58% of participants receiving belimumab 10 mg/kg achieving SRI-4 response [odds ratio (OR) 1.83; 95% confidence interval (CI): 1.30–2.59], compared with 51% of participants receiving belimumab 1 mg/kg (OR: 1.55; 95% CI: 1.10–2.19) and 44% of those receiving placebo.

Corroborating the findings of BLISS-52, BLISS-76 [3] was a Phase III, multicentre, double-blind, placebo-controlled trial that randomised 819 patients with active SLE, mainly in North America and Europe. Participants received belimumab 1 mg/kg (n = 271), 10 mg/kg (n = 273) or placebo (n = 275) plus ST over 76 weeks. At week 52, the SRI-4 response frequencies were 43% with belimumab 10 mg/kg and 41% with belimumab 1 mg/kg, compared to 34% with placebo (*p* = 0.013 for the 10 mg/kg dose and *p* = 0.089 for the 1 mg/kg dose). While response frequencies remained numerically higher at week 76 with both belimumab doses versus placebo, the differences were not statistically significant [3]. Since the 10 mg/kg dose of belimumab consistently yielded improvements based on SRI-4 rates that were significantly greater than those yielded by placebo across both BLISS-52 and BLISS-76, along with an absence of meaningful differences in overall rates of adverse events between the two doses, the 10 mg/kg dose was approved as the standard treating dose.

### 2.3. BLISS-NEA

Building on the success of BLISS-52 and BLISS-76, another Phase III trial was conducted to specifically evaluate belimumab in North East Asian patients with SLE (BLISS-NEA) [4]. This randomised, double-blind trial enrolled 677 patients from China, Japan, and South Korea who received i.v. belimumab 10 mg/kg or placebo plus ST. The results reinforced previous findings, with significantly more patients achieving an SRI-4 response at week 52 in the belimumab group versus placebo (54% versus 40%; OR: 1.99; 95% CI: 1.40–2.82). The study also demonstrated a significant reduction in severe flares and corticosteroid-sparing effects with belimumab. The safety profile remained consistent with earlier trials, with no new concerns identified in this population.

### 2.4. EMBRACE

Building on the need to understand the efficacy of belimumab across diverse populations, the EMBRACE trial, which introduced a modified version of the SRI incorporating the SLE Disease Activity Index 2000 scoring system (SRI-SLEDAI-2K), examined patients of Black African ancestry with SLE [5]. This Phase III/IV randomised, placebo-controlled trial enrolled 448 participants, and while the primary endpoint using this adapted measure at week 52 was not achieved, numerically higher response frequencies were observed in the belimumab 10 mg/kg group compared to placebo (49% versus 42%; OR: 1.40; 95% CI: 0.93–2.11). Notably, patients with high disease activity or renal involvement showed greater benefit. The safety profile remained consistent with previous belimumab trials, with no new concerns identified in this population.

### 2.5. BLISS-SC

Although earlier trials had established the efficacy of i.v. belimumab at 10 mg/kg, the BLISS-SC trial explored a s.c. route of administration in 839 randomised patients with moderate-to-severe SLE [6]. This randomised, double-blind, placebo-controlled study assessed weekly s.c. belimumab 200 mg versus placebo over 52 weeks. The trial met its primary endpoint, with 61% of belimumab-treated patients achieving an SRI-4 response at week 52 compared to 48% in the placebo group (OR: 1.68; 95% CI: 1.25–2.25). In line with BLISS-NEA [4], this trial showed belimumab benefit in reducing severe flare rates and facilitating steroid tapering. BLISS-SC established s.c. belimumab as an alternative to i.v. administration.

### 2.6. BLISS-LN

The two-year, randomised, controlled trial of belimumab in lupus nephritis (BLISS-LN) represented a pivotal advance in lupus nephritis (LN) treatment [7]. This Phase III, multinational study randomised 448 patients with active LN to either receive belimumab (10 mg/kg) or placebo alongside ST. At week 104, the primary efficacy renal response was achieved in 43% of patients receiving belimumab compared to 32% of patients receiving placebo. The trial demonstrated significant benefits in reducing renal-related events or death [hazard ratio (HR) 0.51; 95% CI: 0.34–0.77] and established the efficacy of belimumab in active LN, leading to its approval for this indication.

### 2.7. PLUTO

Following adult efficacy data, belimumab was studied in paediatric SLE. The PLUTO trial investigated i.v. belimumab in children with SLE aged 5–17 years [56]. This Phase II randomised, double-blind, placebo-controlled study enrolled 93 participants. The primary endpoint using SRI-4 demonstrated a 53% response frequency with belimumab versus 44% with placebo at week 52, although the difference was not significant (OR: 1.49; 95% CI: 0.64–3.46). Safety analysis revealed fewer serious adverse events with belimumab (17% versus 35%), leading to regulatory approval.

### 2.8. BLISS-BELIEVE

The BLISS-BELIEVE trial [57] explored whether combining the established efficacy of belimumab with rituximab, which alone had failed primary endpoints in clinical trials [61,62], could enhance outcomes in SLE. This Phase III, double-blind study randomised 292 patients to either receive belimumab with rituximab, belimumab with placebo, or belimumab with ST. The primary endpoint of disease control (SLEDAI-2K ≤ 2 without immunosuppressants and glucocorticoids at a dose ≤ 5 mg/day of a prednisone equivalent) at week 52 was achieved in 19% of patients who received the combination of belimumab and rituximab versus 17% receiving belimumab and placebo. Although the combination did not show statistically significant superiority, it demonstrated deeper reductions in B cell counts and improvements in serological markers.

### 2.9. OBSErve

As belimumab has become increasingly used in clinical practice, several important real-world observational studies have helped corroborate its effectiveness. The OBSErve programme examined outcomes up to 24 months across six countries, with the US study being the largest (n = 501) [63], followed by the studies in Germany (n = 102) [64], Argentina (n = 81) [65], Spain (n = 64) [66], Switzerland (n = 53) [67], and Canada (n = 52) [68]. At the 6-month mark, over 70% of patients achieved ≥20% improvement in physician-assessed disease activity, mean SELENA-SLEDAI scores decreased significantly, and substantial steroid-sparing effects were observed, with mean prednisone doses typically halving [63,64,65,66,67,68]. Importantly, healthcare resource utilisation generally decreased across the OBSErve studies. Additional data from other real-life experiences in Greece [69,70], Sweden [71,72], Italy [73,74,75], Spain [76], and Brazil [77] reported concordant efficacy results on multiple efficacy outcomes, including SLEDAI-2K scores, PGA, flare rates, attainment of low disease activity (LDA) and remission, and patient-reported outcomes. Moreover, these studies helped to identify predictors of treatment response, including shorter disease duration and low or no baseline damage as predictors of better belimumab efficacy [71,75].

## 3. Results from Post Hoc Analyses of the BLISS Trials

### 3.1. Efficacy Endpoints and Outcome Measures

#### 3.1.1. SRI-4

The SRI-4 [78] is defined as ≥ 4-point reduction from baseline in the SELENA-SLEDAI score [58], or in the SLEDAI-2K score [79] in later adaptations [5], no worsening in PGA defined as an increase of ≥0.3 points on the SELENA-SLEDAI PGA 0–3 scale [80,81], and no new British Isles Lupus Assessment Group (BILAG) [82] A organ domain score or 2 new BILAG B organ domain scores compared with baseline at the time of assessment. The SRI-4 was the primary endpoint of the Phase III trials of belimumab in SLE, and was met in all trials, with the exception of EMBRACE [5]. In BLISS-52 [2], BLISS-76 [3], BLISS-NEA [4], and BLISS-SC [6], analysis of the individual components of the SRI-4 demonstrated significant improvements in favour of belimumab for all three parameters, i.e., ≥4-point reduction in SELENA-SLEDAI score, no worsening in PGA, and no new BILAG A or two new BILAG B organ domain scores. These results were confirmed when analysing pooled data from the five Phase III BLISS trials, where belimumab yielded an OR of 1.67 (95% CI: 1.36–2.03) for SRI-4 response at week 52 compared with placebo [13] (Table 2). Another analysis comparing data from the paediatric PLUTO trial [56] and the adult Phase III trials of i.v. belimumab [2,3,4,5] found similar SRI-4 response rates across the two populations, demonstrating consistent efficacy and safety of belimumab in paediatric and adult patients with SLE [11]. SRI-4 response in the BLISS trials has been linked to improvements in different outcomes, including glucocorticoid need, flare rates, and patient-reported fatigue, strengthening its value as a clinically meaningful primary endpoint in SLE [23,25].

#### 3.1.2. BICLA

The BILAG-based Combined Lupus Assessment (BICLA) is another outcome measure, which has been used as a primary and secondary endpoint in several SLE trials, including the anifrolumab trials [83,84,85]. The BICLA definition requires BILAG 2004 improvement in all affected domains at baseline (A domain scores to B, C, or D; and B domain scores to C or D) and no worsening of disease activity in other organ systems (defined as ≥1 new A domain score(s) or ≥2 new B domain scores); no increase in the SLEDAI-2K score from baseline; no numeric increase in the PGA score ≥ 0.3 points from baseline (scale range: 0–3); and no treatment failure [86]. In a recent post hoc analysis of the BLISS-52, BLISS-76, BLISS-NEA, BLISS-SC, and EMBRACE SLE populations, significant greater proportions of BICLA responders were seen among belimumab-treated patients compared with placebo recipients, with differences starting from week 8 and being maintained through the 52-week observation period, and with an OR of 1.47 (95% CI: 1.25–1.72) in favour of belimumab over placebo at week 52 [17] (Table 2). BICLA has distinct strengths and limitations. Its criteria require documented improvement in all baseline-affected domains according to the BILAG index [82], ensuring clinically meaningful progress across multiple systems. Unlike SRI-4, BICLA does not require complete resolution of a manifestation; substantial partial improvement based on BILAG scoring is sufficient [78,87]. In comparison, SRI-4 primarily focuses on the SLEDAI score [78] and mandates complete resolution of at least one manifestation in a SLEDAI organ domain, corresponding to an improvement of ≥4 points [78]. These differences in approach have led to divergent response evaluations in clinical trials [88], highlighting their complementary nature as outcome measures.

#### 3.1.3. LLDAS and DORIS Remission

Attainment of LDA and, if possible, remission, is the recommended target of SLE treatment [89,90]. Lupus Low Disease Activity State (LLDAS) [91,92] and Definition Of Remission In SLE (DORIS) remission [93,94] are two widely adopted sets of criteria for these targets. LLDAS is defined as a SLEDAI-2K score ≤ 4 with no major organ activity or fever and no new activity in any descriptor since the previous assessment, a PGA score ≤ 1 (scale: 0–3), and concomitant prednisone (or equivalent) dose ≤7.5 mg/day, whereas DORIS remission is more stringent, requiring a clinical SLEDAI-2K score of 0 (SLEDAI-2K score after elimination of the serological descriptors), PGA < 0.5 (scale: 0–3), and prednisone (or equivalent) ≤ 5 mg/day. LLDAS and DORIS remission were not included as efficacy endpoints in the belimumab trials, which were conducted before the introduction of disease control targets. Nevertheless, the relationship between LLDAS and DORIS remission attainment and belimumab treatment was subsequently investigated in a series of post hoc analyses of the BLISS trials [8,9,10,12,13,14] (Table 2). A recent meta-analysis of pooled data from five Phase III trials (BLISS-52, BLISS-76, BLISS-NEA, BLISS-SC, EMBRACE) showed significant differences in attainment of DORIS remission (as early as week 28) and LLDAS (as early as week 8) in favour of belimumab, with significantly higher proportions of belimumab-treated compared with placebo-recipients attaining DORIS remission [risk ratio (RR): 1.51; 95% CI 1.15–1.99] and LLDAS (RR: 1.74; 95% CI: 1.44–2.12) at week 52 [14] (Table 2). The same study showed that patients treated with belimumab were significantly more likely to reach sustained (i.e., at ≥2 consecutive visits) and maintained (i.e., through week 52) DORIS remission and LLDAS compared with placebo recipients [14]. Real-world experiences have demonstrated consistent findings, yet higher attainment rates [69,75,95].

#### 3.1.4. Patient-Reported Outcome Measures

Patients with SLE experience significant impairments in HRQoL compared with the general population and with patients with other chronic conditions [96]. Post hoc analyses of the BLISS trials have highlighted an association between LDA/remission and greater patient-reported health states [12,31], and between decreased disease activity and HRQoL [26,27]. However, a proportion of patients reports poor HRQoL even in the setting of an adequate response to therapy assessed with physician-centred activity measures [18,20]. The effect of belimumab on patient-reported outcome measures (PROMs) was assessed in an early post hoc analysis of the BLISS-52 and BLISS-76 trials [24], using the SF-36 [59], Functional Assessment of Chronic Illness Therapy (FACIT)-Fatigue [97], and EQ-5D questionnaires [98]. In that study, greater improvements in SF-36 PCS and mental component summary (MCS) were observed at week 52 in patients receiving belimumab compared with placebo [24]. Similarly, improvement in FACIT-Fatigue scores at week 52 were greater in the belimumab arms compared with the placebo arm [24] (Table 2). Consistently, other studies highlighted a lower probability of experiencing poor HRQoL outcomes using the SF-36 and FACIT-Fatigue scales in patients on belimumab plus ST versus patients receiving ST alone [20,21] (Table 2). Another post hoc analysis of the same trials focused on the proportion of patients experiencing EQ-5D full health state (FHS), defined as a response of no problem across all five EQ-5D dimensions (i.e., EQ-5D index score corresponding to the maximum value of 1.000) [19] (Table 2). That study showed higher EQ-5D FHS frequencies among patients receiving the licenced belimumab dose versus patients receiving placebo [19]. A subsequent study found similar results using less stringent EQ-5D values, i.e., EQ-5D index scores above 0.800 [30]. More recently, a machine learning-powered analysis applied to the same dataset identified older age, female sex, non-Asian ancestry, high disease activity, and low urinary protein to creatinine ratio (UPCR) to be associated with a lack of EQ-5D FHS experience at baseline and after the 52-week trial intervention. Importantly, high baseline EQ-5D-3L index scores constituted the strongest predictor of EQ-5D FHS reports at week 52 [33]. Overall, these findings provide support for the integration of PROMs into the global evaluation of patients with SLE, complementing traditional clinical assessments and ensuring that treatment outcomes better reflect the patient’s lived experience and overall well-being. Results of post hoc analyses addressing the effect of belimumab on PROMs are summarised in Table 2.

### 3.2. Belimumab Efficacy Across Organ Manifestations

#### 3.2.1. Lupus Nephritis

The BLISS-LN trial demonstrated a beneficial effect of belimumab as an add-on therapy in patients with active LN [7]. An early post hoc analysis of the BLISS-LN by Rovin et al. showed that add-on belimumab was most effective in yielding primary efficacy renal response [PERR; UPCR ≤ 0.7 g/g, estimated glomerular filtration rate (eGFR) no worse than 20% below the pre-flare value or ≥60 mL/min/1.73 m^2^, and no rescue therapy for treatment failure] and complete renal response (CRR; UPCR < 0.5 g/g, eGFR no worse than 10% below the pre-flare value or ≥90 mL/min/1.73 m^2^, and no rescue therapy) in patients with proliferative LN and a baseline UPCR under 3 g/g, compared with patients with UPCR equal or above 3 g/g or membranous LN [38]. Moreover, belimumab significantly improved the long-term outcomes including chronic kidney disease, risk of renal flare, and risk of kidney-related events or death (defined as occurrence of one of the following: end-stage kidney disease, doubling of serum creatinine, increased proteinuria and/or impaired kidney function, kidney disease-related treatment failure, or all-cause death) [38]. Another post hoc analysis of the BLISS-LN and BLISS-LN open label extension by Malvar et al., conducted on a subgroup of patients who underwent per-protocol repeat kidney biopsy after a median follow-up of 43.6 months after the end of the study, showed that add-on belimumab on top of mycophenolate mofetil (MMF) was associated with complete histological response, i.e., a National Institutes of Health (NIH) Activity Index (AI) score of 0 [39]. To this end, another study found that benefits of belimumab on kidney outcomes were similar for newly diagnosed LN and relapsed LN patients, irrespective of the glucocorticoid dose in the initial phase of LN treatment [37].

Although patients with severe active LN were excluded from the initial belimumab Phase III studies, post hoc analyses of these studies demonstrated benefits from belimumab on kidney outcomes in SLE patients with evidence of active kidney disease based on BILAG (summarised in Table 3) [43,44,45,99]. Interestingly, a protection against de novo renal SLE conferred from 1 mg/kg i.v. (HR: 0.38; 95% CI: 0.20–0.73) and 200 mg s.c. (HR: 0.69; 95% CI: 0.54–0.88) but not 10 mg/kg i.v. belimumab was demonstrated in patients with SLE and no prior nephritis history [43], which along with real-world observations of nephritis occurrence in SLE patients under treatment [100,101] and documentation of rapid reductions in IL-10 levels upon belimumab treatment commencement [102] has resulted in speculations about effects of belimumab on B cells with regulatory properties [43,46]. Collectively, further investigation of the effects of lower belimumab doses of belimumab appears warranted, especially for the treatment of SLE patients with low or absent renal activity.

In conclusion, belimumab on top of ST adds benefit in attaining renal response and helps improve long-term kidney outcomes by enhancing renal protection and reducing the risk of flares in patients with active lupus nephritis, especially if initiated early.

Results of post hoc analyses of the BLISS trials with focus on LN are reported in Table 3.

#### 3.2.2. Neuropsychiatric SLE

Patients with active severe central nervous system disease were excluded from belimumab trials, thus limiting analyses on neuropsychiatric SLE (NPSLE). A post hoc analysis of data from BLISS-52, BLISS-76, BLISS-NEA, BLISS-SC, and EMBRACE identified predictors of NPSLE flares in patients with active disease yet no severe nervous system involvement, assessed with BILAG. Predictors included male sex, prior neuropsychiatric involvement (neuropsychiatric BILAG B–D scores), and higher baseline SDI score. Organ damage in the SDI neuropsychiatric category, particularly cognitive impairment and transverse myelitis, emerged as the strongest determinant of flares [42]. Importantly, belimumab neither reduced nor increased the risk of NPSLE flares. Complementary findings by Nikolopoulos et al. revealed that NPSLE patients reported significantly lower HRQoL and higher fatigue levels than those without neuropsychiatric involvement, regardless of current organ manifestations [29], underscoring the debilitating impact of NPSLE on patients’ well-being. Further analysis demonstrated that neuropsychiatric involvement is associated not only with damage in the nervous system but also damage in other organ systems [41].

#### 3.2.3. Skin and Musculoskeletal Involvement

Several studies have explored the efficacy of belimumab across musculoskeletal and cutaneous SLE manifestations. A post hoc analysis of the BLISS-52 and BLISS-76 trials highlighted that belimumab-treated patients had significant improvements in the cutaneous as well as musculoskeletal domains, assessed with either BILAG or SELENA-SLEDAI [40]. In addition, treatment response in musculoskeletal and mucocutaneous organ system domains has been linked with improvements in SF-36 scores in a recent post hoc analysis [27]. In a meta-analysis of six belimumab trials, Kneeland et al. investigated the effect of belimumab on cutaneous manifestations after 52 weeks of treatment, defined as a decrease in the cutaneous domain from a baseline BILAG A to a BILAG score of B through E, and from a baseline BILAG B to a BILAG C through E score in patients with cutaneous manifestations at baseline. In this study, belimumab use yielded an OR of 1.44 (95% CI: 1.20–1.74) for attainment of cutaneous clinical response at week 52, with significant differences observed as early as week 20 from baseline (OR: 1.35; 95% CI: 1.01–1.81) [47]. In addition, the same study showed a protective effect of belimumab against cutaneous flares [47]. In another recent post hoc analysis of five Phase III trials of belimumab, belimumab was superior to placebo in inducing mucocutaneous improvement at week 52, measured by BILAG (OR: 1.29; 95% CI: 1.07–1.57) and by SLEDAI-2K (OR: 1.37; 95% CI: 1.16–1.62), as well as in inducing sustained (≥2 consecutive visits, maintained through week 52) mucocutaneous improvement by BILAG (HR: 1.23; 95% CI: 1.07–1.41) and by SLEDAI-2K (HR: 1.24; 95% CI: 1.17–1.31) [36]. Collectively, these findings indicate that belimumab significantly improves cutaneous and musculoskeletal manifestations and reduces the risk of flares, supporting its use in patients with mucocutaneous or musculoskeletal disease to achieve sustained organ-specific response.

#### 3.2.4. Organ Damage

Organ damage is a major determinant of morbidity and mortality in SLE patients [104,105], and thus prevention of damage accrual represents the overarching goal of treatment [89]. In the original Phase III belimumab trials, no difference emerged between belimumab and placebo in Systemic Lupus International Collaborating Clinics (SLICC)/American College of Rheumatology (ACR) Damage Index (SDI) scores after 52 weeks, which may be expected given the short time of observation [2,3,4,5,6]. However, the impact of belimumab on organ damage accrual was assessed in the long-term open label study extensions of the Phase III belimumab trials [50,106,107,108,109]. In these studies, patients assigned to the belimumab arm continued to receive belimumab, and patients previously receiving placebo were switched to receive belimumab. Belimumab proved effective and safe over up to 8 years of follow-up [107], yielding minimal organ damage accrual assessed using the SDI, with lack of SDI increase documented in up to 88% of patients [107]. In an analysis of pooled data from BLISS-52 and BLISS-76 open label studies, 343 out of 403 (85%) patients had no change in SDI score from baseline after a 6-year follow-up. Damage accrual appeared to be irrespective of baseline organ damage [50]. In another open label study, including 142 South Korean and Japanese patients from both BLISS-NEA and BLISS-SC, only 4 out of 117 patients from BLISS-NEA and 2 out of 25 patients from BLISS-SC experienced SDI worsening compared to baseline over a 7-year follow-up [108]. Two other studies comparing patients receiving belimumab from open label extension studies and propensity matched patients from the Toronto Lupus Clinic Cohort treated with ST found that patients treated with belimumab had 60–61% lower risk of SDI progression compared with patients receiving real-world ST over a 5-year follow-up (HR: 0.39–0.40) [48,49].

With specific regard to kidney damage, a post hoc analysis of the BLISS-LN trial showed that patients receiving belimumab were less likely to experience a sustained decline in eGFR compared to placebo, yielding an OR of 0.29 (95% CI: 0.13–0.68) for 30% decline and an OR of 0.25 (95% CI: 0.08–0.78) for 40% decline [38].

In conclusion, long-term treatment with belimumab is associated with reduced accrual of organ damage, highlighting its role in modifying disease course and supporting prolonged therapy, especially in patients at risk of accumulating organ damage over time.

#### 3.2.5. Serologically Active Disease

Subgroup analyses of the early Phase II and subsequent Phase III belimumab trials provided evidence of the efficacy of belimumab in mitigating serological activity. In the BLISS-76 trial [2], belimumab treatment resulted in sustained reductions in anti-dsDNA antibody levels and increases in C3/C4 levels from week 8 through week 76, and similar beneficial effects were described in EMBRACE [5]. Belimumab efficacy was shown to be greater in serologically active patients compared with counter subgroups, i.e., patients with positive anti-dsDNA levels and/or low C3/C4 levels; this was documented across several post hoc analyses of the BLISS trials, using different efficacy outcome measures, including SRI-4 [110,111,112], BICLA [17], LLDAS [14,16], DORIS remission [14,16], and PROMs [19,111]. Two post hoc analyses [111,112] showed enhanced therapeutic benefit of belimumab compared with ST alone in patients with higher disease activity, anti-dsDNA positivity, and low complement levels at baseline, holding true for both the i.v. [111] and the s.c. [112] administration. Also, patients with either low complement or positive anti-dsDNA, rather than both, attained SRI-4 response to a greater extent with belimumab than with placebo at week 52 [110]. In a meta-analysis of five Phase III trials of belimumab, in patients with positive anti-dsDNA antibody levels and patients with positive anti-dsDNA antibody levels and/or low complement C3 and/or C4 levels, belimumab conferred significantly greater BICLA response rates compared with placebo after 52 weeks of follow-up. Another meta-analysis of the same trials found that belimumab conferred greater efficacy in inducing LLDAS or DORIS remission among patients with serologically active SLE at baseline compared with counter subgroups [14].

Longitudinal changes in anti-dsDNA antibody and C3/C4 levels have also been associated with response to belimumab, as a greater decline in anti-dsDNA and a greater increase in C3 and C4 levels was seen in SRI-4 responders compared with non-responders from baseline through week 52 [113]. Conversely, increase or lack of decrease in anti- dsDNA levels heralded subsequent severe disease flares [46]. Along similar lines, another post hoc analysis of five RCTs of belimumab showed that negative anti-dsDNA or normal/high C3/C4 levels were associated with enhanced protection against severe SLE flares or renal flares [114].

Among other serological markers, anti-Smith (Sm) antibody positivity has been shown to be associated with enhanced clinical response to belimumab [102]. In a post hoc analysis of the BLISS-52 and BLISS-76 trials, higher proportions of EQ-5D FHS at week 52 were seen in anti-Sm positive versus negative patients who received belimumab the approved dose of belimumab, while no such difference was observed among placebo recipients [19].

Overall, these findings support the use of serological activity to guide belimumab initiation and identify patients likely to derive optimal benefit.

#### 3.2.6. Trajectories of Disease Evolution

Trajectory modelling has recently gained traction as a valuable tool in studying chronic diseases like SLE by helping predict disease progression and identify subgroups with distinct patterns, thereby disentangling disease heterogeneity and supporting personalised therapeutic decisions [115]. In a post hoc analysis of three clinical trials of belimumab, we modelled clinical, laboratory, and patient-reported data in combined analysis and identified four latent classes with differential evolution of disease activity and patient-reported fatigue, with those features having been selected in an unsupervised fashion. These findings highlight the diverse nature of SLE and underscore the importance of incorporating patient-reported health experience in disease assessment and monitoring [35].

### 3.3. Drug-Related Aspects

#### 3.3.1. Synergy with Antimalarial Agents

Accumulating evidence supports the use of antimalarial agents (AMA) in combination with belimumab for increased likelihood of favourable responses to this biological therapy. In a recent meta-analysis of five belimumab RCTs, BICLA response rates were greater in belimumab-treated when combined with AMA [17]. Accordingly, other post hoc analyses have demonstrated higher frequencies of EQ-5D FHS reports with the combined regimen [19] and greater protection against development of renal flares [44]. In another post hoc analysis of BLISS-52 and BLISS-76 data, it was shown that IV belimumab 10 mg/kg induced significant decreases in IgG and IgA anticardiolipin (aCL) antibody levels only in the subgroup of patients who were concomitantly treated with AMA [116]. A similar observation was made in a post hoc analysis of the BLISS-SC trial, which reported significantly greater decreases in IgM aCL and IgA anti-β2-glycoprotein I (anti-β2-GPI) levels with belimumab compared with placebo only in the subgroup of patients who were concomitantly receiving AMA [117]. These findings may be partly explained by the observed association between AMA use and lower B-cell activating factor (BAFF) levels [118], thereby enhancing the pharmacodynamic effects of belimumab, which directly targets soluble BAFF. While a consolidated mechanistic explanation has not been provided yet, these studies suggest a synergy between belimumab and AMA, with AMA potentiating the effect of belimumab.

#### 3.3.2. Safety and Side Effects

Belimumab is generally safe and well-tolerated, with an overall positive risk-benefit profile. In a meta-analysis of data from one Phase II and five Phase III belimumab trials in adult patients with SLE, the incidence of adverse events (AEs), serious AEs (SAEs), severe AEs, AEs of special interest (AESI), and mortality over a 52-week study period were similar between belimumab and placebo [119]. Reassuring results were obtained across other populations, including paediatric patients [56], older adults [120], and patients with LN [7].

Among the most frequently observed side effects is the occurrence of infections and infestations, especially of the respiratory and urinary tract, which have been reported in multiple post hoc analyses of RCTs [119,121], yet with comparable rates between belimumab and placebo. In addition, in the Phase IV BASE trial investigating the incidence of all-cause mortality and AESI with belimumab versus placebo, a higher incidence of serious depression (placebo: 0.1% [n = 1/2001]; belimumab: 0.4% [n = 7/2002]) and serious suicidal ideation or self-injury (placebo: 0.25% [n = 5/2001]; belimumab: 0.75% [n = 15/2002]) was found in patients receiving belimumab compared with placebo [122]. A higher incidence of depression was also found in another meta-analysis of data from six belimumab trials, raising concerns on the use of this drug in patients with a previous history of psychiatric disorder [119]. However, in long-term follow-up studies, depression, suicidal ideation, and/or self-injury rates decreased with belimumab. These findings highlight the importance of close monitoring, especially during the early stages of treatment.

## 4. Conclusions

In summary, over the past decade, more than 50 post hoc analyses of clinical trials of belimumab have been conducted and have advanced our understanding of the use of the drug, along with deepening our overall understanding of SLE. By exploring a broad spectrum of trial endpoints, these studies have provided valuable insights that may guide the design of future clinical trials and support the development of new therapeutic strategies. In addition to data from randomised settings, emerging real-world evidence has further confirmed the clinical benefits of belimumab in routine practice, often showing higher attainment rates of LLDAS and remission. Notably, several studies suggest that earlier initiation of belimumab, particularly in serologically active patients, may enhance long-term outcomes by promoting faster disease control and reducing cumulative exposure to glucocorticoids [69,71,75,123]. On the other hand, evidence on treatment discontinuation remains limited, with only a small real-world retrospective study suggesting that discontinuing belimumab treatment after achieving disease stability may be associated with a subsequent increase in disease activity and higher glucocorticoid dosages [124]. Therefore, tapering or discontinuation of belimumab should be undertaken with caution, careful patient selection and individualised risk assessment. The findings reinforce the efficacy of belimumab in treating specific disease manifestations, preventing disease flares, including renal flares, and reducing long-term exposure to glucocorticoids and organ damage accrual. Additionally, observed improvements in patient-reported outcomes have underscored the broader impact of belimumab on disease burden and highlighted the importance of incorporating patient-reported HRQoL in SLE surveillance, promoting more patient-centred and holistic management approaches [125,126]. Collectively, these studies offer advanced and clinically relevant insights into both the natural history and the therapeutic landscape of SLE.

## Figures and Tables

**Table 1:** Summary of belimumab trials.

Study	Setting	Population	Study Groups	Primary Endpoint	Primary Endpoint Achieved?
Wallace et al. 2009 [55]	Phase II RCT	Adults with active SLE (N = 449)	belimumab 1, 4 and 10 mg/kg i.v.; placebo	Percent change in SELENA-SLEDAI at week 24	No
Navarra et al. 2011 [2]	Phase III RCT	Adults with active SLE (N = 867)	belimumab 1 and 10 mg/kg i.v.; placebo	SRI-4 at week 52	Yes
Furie et al. 2011 [3]	Phase III RCT	Adults with active SLE (N = 819)	belimumab 1 and 10 mg/kg i.v.; placebo	SRI-4 at week 52	Yes
Stohl et al. 2017 [6]	Phase III RCT	Adults with active SLE (N = 836)	belimumab 200 mg s.c.; placebo	SRI-4 at week 52	Yes
Zhang et al. 2018 [4]	Phase III RCT	Adults with active SLE (N = 677)	belimumab 10 mg/kg i.v.; placebo	SRI-4 at week 52	Yes
Furie et al. 2020 [7]	Phase III RCT	Adults with active LN (N = 448)	belimumab 10 mg/kg i.v.; placebo	PERR at week 104	Yes
Brunner et al. 2020 [56]	Phase II RCT	Children with active SLE (N = 93)	belimumab 10 mg/kg i.v.; placebo	SRI-4 at week 52	No
Ginzler et al. 2022 [5]	Phase III/IV RCT	Adults with active SLE (N = 448)	belimumab 10 mg/kg i.v.; placebo	SRI-SLEDAI-2K at week 52	No
Aranow et al. 2024 [57]	Phase III RCT	Adults with active SLE (N = 263)	rituximab 1000 mg i.v. or placebo on top of belimumab 200 mg s.c.	SLEDAI-2K ≤ 2 without immunosuppressants and glucocorticoids at a dose ≤ 5 mg/day of a prednisone equivalent) at week 52	No

i.v.: intravenous; LN: lupus nephritis; PERR: primary efficacy renal response; RCT: randomised controlled trial; s.c.: subcutaneous; SELENA-SLEDAI: Safety of Estrogens in Lupus Erythematosus: National Assessment version of the Systemic Lupus Erythematosus Disease Activity Index; SLE: systemic lupus erythematosus; SLEDAI-2K: SLE Disease Activity Index 2000; SRI-4: SLE Responder Index 4.

**Table 2:** Efficacy of belimumab according to outcome measures in post hoc analyses of clinical trials of belimumab.

Author, Year	Trial (s)	Population *	Comparator	Main Findings
SRI-4				
Brunner et al., 2021 [11]	PLUTO, BLISS-52, BLISS-76, BLISS-NEA, EMBRACE	93 paediatric; 2250 adults (N = 2343)	Adult versus paediatric	Comparable SRI-4 response rates in children (52.8%) and adults (43.2–57.6%) treated with belimumab
Neupane et al. 2023 [13]	BLISS-52, BLISS-76, BLISS-NEA, BLISS-SC, EMBRACE	N = 3080	Belimumab versus ST alone	Belimumab yielded greater SRI-4 response rates (OR: 1.7)
BICLA				
Parodis et al. 2025 [18]	BLISS-52, BLISS-76, BLISS-NEA, BLISS-SC, EMBRACE	N = 2800	Belimumab versus ST alone	Belimumab yielded greater BICLA response rates (OR: 1.5)
LLDAS				
Oon et al. 2019 [8]	BLISS-52, BLISS-76	N = 1684	Belimumab versus ST alone	Belimumab yielded greater LLDAS attainment rates (OR: 2.3)
Parodis et al. 2019 [9]	BLISS-52, BLISS-76	N = 563	NA	SDI > 0 had a negative impact on LLDAS attainment among patients receiving belimumab (OR: 0.4)
Parodis et al. 2024 [14]	BLISS-52, BLISS-76, BLISS-NEA, BLISS-SC, EMBRACE	N = 3086	Belimumab versus ST alone	Belimumab yielded greater LLDAS attainment rates (RR: 1.7)
Depascale et al. 2025 [15]	BLISS-SC	N = 796	NA	Three distinct endotypes based on B cell subset and serological profiles displayed distinct belimumab benefit for sustained LLDAS
DORIS remission				
Parodis et al. 2019 [9]	BLISS-52, BLISS-76	N = 1684	Belimumab versus ST alone	Belimumab yielded greater sustained DORIS remission attainment rates (OR: 2.1)
Parodis et al. 2024 [14]	BLISS-52, BLISS-76, BLISS-NEA, BLISS-SC, EMBRACE	N = 3086	Belimumab versus ST alone	Belimumab yielded greater DORIS remission attainment rates (RR: 1.5)
Depascale et al. 2025 [15]	BLISS-SC	N = 796	NA	Three distinct endotypes based on B cell subset and serological profiles displayed distinct belimumab benefit for sustained DORIS remission
PROMs				
Strand et al. 2014 [24]	BLISS-52, BLISS-76	N = 1684	Belimumab versus ST alone	Belimumab yielded greater improvements in PCS, SF-36 vitality domain, and FACIT-F scores
Furie et al. 2014 [23]	BLISS-52, BLISS-76	N = 1684	SRI-4 responders versus non-responders	Responders had greater SF-36 PCS (4.9 vs. 2.6), MCS (4.4 vs. 1.7) and FACIT-F (5.2 vs. 3.0) score change
van Vollenhoven et al. 2018 [25]	BLISS-SC	N = 833	SRI-4 responders versus non-responders	Responders had greater mean change in FACIT-F scores (35.6 vs. 18.7%)
Borg et al. 2021 [21]	BLISS-52, BLISS-76	N = 1684	Belimumab versus ST alone	Belimumab protected against adverse SF-36 general health (OR: 0.8) and FACIT-F < 30 (OR: 0.8)
Gomez et al. 2021 [20]	BLISS-52, BLISS-76	N = 760	Belimumab versus ST alone	Belimumab protected against adverse SF-36 physical functioning (OR: 0.6) and FACIT-F (OR: 0.5)
Gomez et al. 2020 [22]	BLISS-52, BLISS-76	N = 1684	NA	Overweight and obesity were associated with PCS, FACIT-F and EQ-5D scores
Lindblom et al. 2021 [19]	BLISS-52, BLISS-76	N = 1684	Belimumab versus ST alone	Belimumab yielded higher EQ-5D FHS frequencies (26.1 vs. 19.4%; OR: 1.6)
Rendas-Baum et al. 2021 [26]	BLISS-52, BLISS-76, BLISS-SC	N = 2520	Responders versus non-responders	Improvement in PGA and BILAG scores was associated in higher improvement in FACIT-F scores (−4.4 vs. −2.2)
Emamikia et al. 2022 [12]	BLISS-52, BLISS-76	N = 1684	NA	Remission and LLDAS contributed to clinically important HRQoL benefit in a time-dependent manner
Lindblom et al. 2022 [34]	BLISS-52 and BLISS-76 open label studies	N = 973	NA	EQ-5D FHS was associated with reduced hazard to accrue organ damage (HR: 0.6)
Borg et al. 2023 [32]	BLISS-52, BLISS-76	N = 1684	NA	Obesity was associated with impaired mobility (OR: 2.1), affecting HRQoL outcomes
Jesus et al. 2024 [31]	BLISS-52, BLISS-76	N = 1684	LDA/remission attainers versus non attainers	Patients achieving SLE-DAS remission and LDA had greater improvements in SF-36 and FACIT-F scores
Parodis et al. 2024 [35]	BLISS-52, BLISS-76, BLISS-SC, EMBRACE	N = 2868	NA	Four classes with distinct disease trajectories upon treatment initiation were identified, based on physician-and patient-reported (FACIT-F) outcome measures
Rendas-Baum et al. 2024 [27]	BLISS-52, BLISS-76, BLISS-SC, EMBRACE	N = 1066	Responders versus non-responders	Responders had greater improvement in SF-36 and FACIT-F scores across several SLEDAI organ domains
Botto et al. 2025 [33]	BLISS-52, BLISS-76	N = 1642	NA	Older age, female sex, non-Asian ancestry, high disease activity and low UPCR were associated with a lack of EQ-5D FHS experience at baseline and week 52
Parodis et al. 2025 [18]	BLISS-52, BLISS-76, BLISS-SC, EMBRACE	N = 2406	NA	Despite LLDAS or DORIS remission, substantial proportions of SLE patients experienced poor HRQoL

BLISS: Study of Belimumab in Subjects with SLE; BICLA: British Isles Lupus Assessment Group (BILAG)-based Combined Lupus Assessment; DORIS: Definitions of Remission in SLE; EMBRACE: Efficacy and Safety of Belimumab in Black Race Patients with SLE; EQ-5D: EuroQol five dimensions questionnaire; FACIT-F: Functional Assessment of Chronic Illness Therapy Fatigue questionnaire; FHS: full health state; HRQoL: health-related quality of life; LLDAS: Lupus Low Disease Activity Status; MCS: mental component summary; NA: not applicable; OR: odds ratio; PCS: physical component summary; PLUTO: Paediatric Lupus Trial of Belimumab Plus Background Standard Therapy; PROMs: Patient-reported outcome measures; RR: risk ratio; SF-36: Medical Outcomes Survey Short Form; SRI-4: SLE Responder Index 4; ST: standard therapy; UPCR: urine protein to creatinine ratio. * In case of multiple analyses, N refers to the analysis comprising the highest number of patients.

**Table 3:** Summary of post hoc analyses of the BLISS trials analysing organ-specific manifestations of SLE.

Author, Year	Trial (s)	Population	Comparator	Main Findings
Lupus nephritis				
Dooley et al. 2013 [99]	BLISS-52, BLISS-76	N = 1684	Belimumab versus ST alone	Rates of renal flare, renal remission, and improvement in the BILAG and SLEDAI domains; proteinuria reduction favoured belimumab, although the between-group differences were not significant
Rovin et al. 2022 [38]	BLISS-LN	N = 448	Belimumab versus ST alone	Benefits of belimumab in terms of renal response rates were more prominent in patients with PLN compared with MLN; reduced risk of renal flares in belimumab compared with placebo (HR: 0.5); slower eGFR decline in belimumab versus placebo
Anders et al. 2023 [37]	BLISS-LN	N = 448	Belimumab versus ST alone	Benefits of belimumab on kidney outcomes were consistent for newly diagnosed and relapsed patients, irrespective of GC pulses at induction
Malvar et al. 2023 [39]	BLISS-LN	N = 20	Belimumab versus ST alone	Belimumab use increase the possibility of achieving complete histological response in patients undergoing repeat kidney biopsy
Parodis et al. 2023 [43]	BLISS-52, BLISS-76, BLISS-SC, BLISS-NEA, EMBRACE	N = 1844	Belimumab versus ST alone	1 mg/kg i.v. and 200 mg s.c., but not 10 mg/kg i.v., belimumab protected against de novo renal flares
Yu et al. 2023 [103]	BLISS-LN	N = 142	Belimumab versus ST alone	Safety and efficacy of belimumab in the East Asian population were consistent with data in the overall population
Gomez et al. 2024 [44]	BLISS-52, BLISS-76, BLISS-SC, BLISS-NEA	N = 3225	Belimumab versus ST alone	Belimumab conferred enhanced protection against renal flares when co-administered with AMA
Jägerback et al. 2024 [45]	BLISS-52, BLISS-76, BLISS-SC, BLISS-NEA	N = 3225	NA	Current/former renal involvement, hypalbuminaemia, proteinuria and low C3 were associated with impending renal flare
Neuropsychiatric lupus				
Palazzo et al. 2024 [42]	BLISS-52, BLISS-76, BLISS-SC, BLISS-NEA, EMBRACE	N = 3638	Belimumab versus ST	Belimumab use yielded no clear protection against NPSLE flare development
Nikolopoulos et al. 2024 [29]	BLISS-52, BLISS-76, BLISS-SC, EMBRACE	N = 2968	NPSLE versus non NPSLE	NPSLE patients reported lower EQ-5D FHS frequencies, PCS, MCS scores, and severe fatigue
Nikolopoulos et al. 2024 [41]	BLISS-52, BLISS-76, BLISS-SC, BLISS-NEA, EMBRACE	N = 3545	NPSLE versus non NPSLE	NPSLE patients were more likely to develop damage (OR: 2.9) across several organ domains beyond NP
Skin and joint				
Manzi et al. 2012 [40]	BLISS-52, BLISS-76	N = 1684	Belimumab versus ST alone	Greater improvement in BILAG and SLEDAI mucocutaneous and musculoskeletal domains in belimumab-treated patients compared with placebo
Kneeland et al., 2022 [47]	PLUTO, BLISS-52, BLISS-76, BLISS-SC, BLISS-NEA, EMBRACE	N = 1910	Belimumab versus ST alone	OR of 1.4 of cutaneous response attainment at week 52 and OR of 0.5 of cutaneous flare in belimumab-treated patients versus placebo
Grosso et al. 2025 [36]	BLISS-52, BLISS-76, BLISS-SC, BLISS-NEA, EMBRACE	N = 3086	Belimumab versus ST alone	Belimumab was superior to placebo in inducing mucocutaneous improvement at week 52, measured by BILAG (OR: 1.3) and SLEDAI-2K (OR: 1.4)
Organ damage (SDI)				
Bruce et al. 2016 [50]	BLISS-52, BLISS-76 open label extensions	N = 998	NA	Low incidence of organ damage in patients treated with long-term belimumab
Urowitz et al. 2019 [48]	BLISS-52, BLISS-76 open label extensions	N = 99	Belimumab versus ST alone	Smaller SDI increase (mean difference: −0.4) and reduced likelihood of SDI progression (HR: 0.4) in belimumab-treated patients versus ST alone
Urowitz et al. 2020 [49]	BLISS-52, BLISS-76 open label extensions	N = 181	Belimumab versus ST alone	Smaller SDI increase (mean difference: −0.5) and reduced likelihood of SDI progression (HR: 0.4) in belimumab-treated patients versus ST alone

AMA: antimalarial agents; BILAG: British Isles Lupus Assessment Group; BLISS: Study of Belimumab in Subjects with SLE; eGFR: estimated glomerular filtration rate; EMBRACE: Efficacy and Safety of Belimumab in Black Race Patients with SLE; GC: glucocorticoid; HR: hazard ratio; MLN: membranous lupus nephritis; NPSLE: neuropsychiatric SLE; OR: odds ratio; PLN: proliferative lupus nephritis; PLUTO: Paediatric Lupus Trial of Belimumab Plus Background Standard Therapy; SDI: Systemic Lupus International Collaborating Clinics (SLICC)/American College of Rheumatology (ACR) Damage Index; SLEDAI: SLE Disease Activity Index; ST: standard therapy.

## Data Availability

No new data were created or analysed in this study.
